# Driving Adoption and Commercialization of Subunit Vaccines for Bovine Tuberculosis and Johne’s Disease: Policy Choices and Implications for Food Security

**DOI:** 10.3390/vaccines8040667

**Published:** 2020-11-09

**Authors:** Albert I. Ugochukwu, Peter W. B. Phillips, Brian J. Ochieng’

**Affiliations:** Centre for the Study of Science and Innovation Policy, Johnson Shoyama Graduate School of Public Policy, University of Saskatchewan, 101 Diefenbaker Place, Saskatoon, SK S7N 5B8, Canada; peter.phillips@usask.ca (P.W.B.P.); brian.j.ochieng@gmail.com (B.J.O.)

**Keywords:** bovine tuberculosis (bTB), Johne’s disease (JD), food security, livestock industry, subunit vaccines

## Abstract

Infectious animal diseases, such as Johne’s disease (JD) caused by *Mycobacterium avium paratuberculosis* (MAP) and bovine tuberculosis (bTB) caused by *Mycobacterium bovis*, have been a challenge to the livestock industry globally, impacting negatively on animal, human and environmental health, and overall food security. Despite several industry-led and government initiatives and programs aimed at preventing and reducing losses associated with JD and bTB outbreaks, JD has remained endemic in many parts of the world while there have been incidental outbreaks of bTB. While several studies focus on sustainable intensification of food (crop) production as a critical solution to food insecurity, following the existential interconnection between animals, humans and the environment recognized by one health, we frame food security through the lens of animal disease prevention and control, given the importance of livestock products to human health and livelihood. Vaccination has been a popular strategy successfully used in controlling other infectious diseases. The paper focuses on an alternate strategy of two subunit vaccines with companion diagnostics targeted at individual pathogens to attain satisfactory immunological responses for JD and bTB. We examine gaps in vaccine policies, commercialization, and potential strategies that would strengthen animal disease prevention and enhance food security. The potential of public–private partnership in strengthening private sector participation in effective animal disease control and health delivery and the implications for global food security are discussed.

## 1. Introduction

Animal products serve as an important food resource and account for about one-third of human protein consumption globally [1,2]. The health of animals, therefore, has a vital role in human health, sustainable food security, and the achievement of the post-2015 development agenda proposed by the United Nations’ High-Level Panel (http://www.post2015hlp.org). Sustainability of animal agriculture has been affected by a wide range of environmental challenges, especially diseases. We are focused on two endemic diseases—bovine tuberculosis (bTB) and Johne’s disease (JD)—that have become a challenge to policy makers and the livestock industry around the world. These diseases are best examined in the context of the ‘One Health’ (the One Health approach advocates for interdependency of the health of animals, humans and the environment. The ’One Health’ framework is a recent international movement that utilizes a multi-sectoral synergistic approach in promoting human health by reducing risks associated with the “animal-human-ecosystem interface” (http://www.onehealthglobal.net/what-is-one-health/)) methodology for reducing the spread of diseases that are detrimental to animal, humans and the environment.

While some countries have been declared bTB free, others are on the way to managing the disease as a result of certain adopted and ongoing mitigation programs [3]. In Canada, bTB is a federally reportable disease. Several control mechanisms have been initiated by various governments and producer groups to reduce the economic losses associated with animal disease outbreaks. Surprisingly, adoption rates, particularly by the private sector, have remained low. The low rate of adoption has been attributed to several factors, including regulatory policies that ignore incentives and raise the costs of adoption and implementation [4,5].

Vaccination, which has been described as one of the most cost-effective methods of reducing the risk of other livestock diseases and associated economic losses, is widely available for bTB or JD. The case of adoption of the *Escherichia coli* (*E. coli*) vaccine *Econiche* [5] revealed two factors that contributed to low adoption: few market benefits stayed with cattle producers who bear most of the costs of the vaccine, as the greatest proportion of the benefits of vaccination go to consumers and packers/processors along the supply chain; and absence of public or industry-wide incentives or policy mandating the use of *E. coli* vaccine discouraged effective uptake.

Johne’s disease (JD) and bovine tuberculosis (bTB) are both internationally recognized animal diseases that are of significant concern to the livestock industry and food security. It has been estimated that they result in annual losses of US$100 million in Canada [6], while, in the U.S., JD causes an economic loss of about US$100 per cow in every herd that tests positive for JD [7]. The U.S. National Institute for Animal Agriculture concludes that Johne’s disease, an often fatal infection of the small intestine, generates losses of US$250 million in the American dairy cattle industry, where the typical remedy is to slaughter the herd containing infected animals [8]. Estimates for the annual losses caused by these diseases to the livestock producers and cattle industry across nations are high. Recent JD outbreaks resulted in annual losses of US$15.4 million in England [9], US$2500 per 50-animal dairy herd in Canada [10], US$54 million and US$5.4 million in Wisconsin and Pennsylvania of the United States, respectively [11,12], and US$2.1 million in Australia [13]. In addition, beef and dairy industries incur economic losses from JD and bTB through reduced milk production and fertility [14,15], premature culling and reduced slaughter value [16]. There are also losses associated with recalls, international trade and market restrictions, and tainted industry reputation.

Successful eradication and control of these diseases, particularly bTB, has been hampered by wildlife reservoirs and lack of political will, which have made it difficult to develop efficient control strategies [17,18]. Within-herd transmission of bTB has been traced to livestock movement and reservoirs. Once an animal gets infected, the disease spreads through aerosols, direct contacts and sharing of water and feed [19]. In addition, regulators in some jurisdictions often underestimate the effect of zoonotic tuberculosis in animals and humans. In developed countries, some notable hosts, such as buffalo and bison, are husbanded and protected in game reserves, creating a disease reservoir. In developing regions, such as Africa, nomadic pastoralists dominate. Herders and their animals move within and across national borders without any restriction while the animals in most cases share grazing areas and water with indigenous stock, thereby increasing the host spectrum and disease prevalence.

In some countries, such as Canada, existing control measures for bTB include ‘trace-in herds’ practices, which involve the identification via skin tuberculin testing and singling out of animals that have been exposed to infected herds and movement restrictions [20]. Diagnosis and treatment of JD are challenging. Existing diagnostic tests do not detect infected animals until they start showing clinical signs and are shedding *Mycobacterium avium paratuberculosis* (MAP). More so, existing vaccines do not provide full protection [21].

The potential of vaccines in reducing the burden of animal infection has been highlighted [22]. In many cases, effective and cost-saving vaccines are yet to be produced for the livestock industry. A prime example is the case of an *E. coli* vaccine developed to prevent the deadly human pathogen O15:H7. *Econiche*, licensed in Canada in 2008 for use in cattle but currently inactive, was projected to reduce infection by almost 85%. However, adoption was low due to the high unit cost of the vaccine and the prescribed dosage of three injections per animal [22], which some farmers saw as cost ineffective. Borrowing from the lessons on commercialization of the *E. coli* vaccine, one might anticipate that vaccines to combat JD and bTB could be socially beneficial—in that the benefits from use are likely to flow beyond farmers to others in the livestock supply chain and market—but may not be taken up because of the uncertain economic value to producers.

Increased adoption of vaccination for animal disease control complemented with appropriate disease management strategies and sector policies that provide incentives for adoption would create more robust food safety and animal health management. Incorporating new vaccines in addition to or as a replacement for existing policies (e.g., herd depopulation, premature slaughter, and culling) would arguably be an economically superior method for bTB and JD surveillance and control, as it would reduce private and social costs associated with them. Getting the incentives right is the challenge.

This paper focuses on subunit vaccines with companion diagnostics for JD and bTB. We first introduce concepts about vaccine and vaccination and examine gaps in vaccine policies and strategies. We assess commercialization pathways for the subunit vaccines and link them to the classical model of technology adoption decision process. The roles farmers and regulators play in livestock disease management, policy design and incentives are highlighted. We assess the potential of public–private partnerships (PPPs) to increase private sector involvement in efficient disease management. A discussion of the implications of bTB and JD prevention and control for food security concludes the paper.

## 2. Diagnosis and Stages of Bovine Tuberculosis and Johne’s Disease Infection

bTB and JD infection have many similarities. Both diseases progress slowly and show visible clinical signs only at later (advanced) stages, which makes diagnosis very difficult at the early stage. While the *Mycobacteria bovis* (*M. bovis*) that causes bTB is found in the saliva of the infected animal, infection usually starts in the lungs and later spreads to other organs, with clinical signs including anorexia, emaciation, pneumonia, fever, and sometimes enlargement of the lymph nodes [23]. For JD, early clinical signs include chronic diarrhea, weight loss and reduced milk yield [24].

Identified primary maintenance hosts for *M. bovis* include badgers in United Kingdom and Ireland, African Buffalo in South Africa, brush-tailed opossums in New Zealand, white-tailed deer in Michigan in the U.S., bison and elk in Canada, while animals such as goats, pigs, and sheep serve as spillover hosts [25]. On the other hand, ruminant and non-ruminant domesticated animals and wildlife are among a range of hosts for MAP. Although some techniques, such as histopathology [26] and *Mycobacterium tuberculosis* complex (MTBC) mycobacteria [27] have been employed in the diagnosis of bTB, a common standard test has been the single intradermal tuberculin test [28,29], which has high sensitivity and specificity relative to other tests [30]. For JD, reports show that existing diagnostic tests are expensive and low in sensitivity [31].

**Figure 1:**
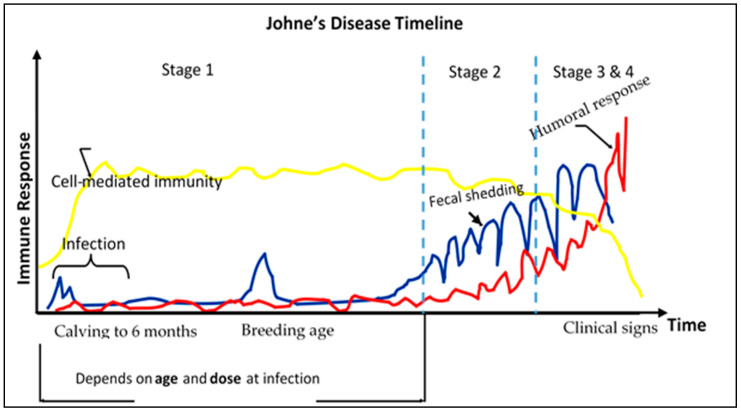
Stage by stage protracted timeline of a bovine infected with Johne’s disease. Source: Adapted from Chiodini et al. [9].

Figure 1 shows an illustration of the protracted nature of Johne’s disease in terms of the number of stages before clinical signs visibly manifest in the animal. Animals can get infected potentially a few months after birth, while clinical signs show at a later stage, usually between 2 to 6 years of age [32]. Manifestation of clinical signs is contingent on two major factors including ‘age at infection’ and ‘dosage of the organism’ [9,33]. Given the time lag between infection and manifestation of clinical signs, a cattle infected at a mature age has low chances of showing clinical signs before the animal is culled [34].

In essence, by the time infection in the animal is identified and confirmed, the carrier animal would have had multiple opportunities to spread the disease to other vulnerable animals within the herd. Although diagnostic tests are currently available, they have proven to be unreliable and ineffective in detecting the presence of infected animals before they reach the stage of super shedding. Efforts to eliminate JD using test-and-cull methods have therefore proven to be unsuccessful [32]. It is difficult to identify and control subclinical cases of JD due to the insidious nature of the infection and elongated incubation period [9].

For bTB, it takes several weeks for the animal to test positive using tuberculin test from the time of infection, while it takes months to years before manifestation of clinical signs [35]. However, the time lag varies from one animal to another.

### 2.1. Vaccination and Animal Vaccines

The use of antibiotics and other chemical drugs on animals have been shown to create residues in food and emergence of antibiotic resistant bacteria in animals [36], suggesting that vaccines have a role in the prevention and treatment of animal diseases, increasing productivity and enhancing food quality [37]. Consumer groups in many countries advocate for restriction of antibiotic use in animals. Unlike antibiotics, vaccination has been identified as the most cost-effective way to eradicate infectious diseases, prevent and/or reduce clinical signs after infection [38] and lower residues in food stuffs [39]. (Infectious diseases in humans and animals have some similarities, particularly in terms of pathogenesis [40]. However, the purpose of vaccination differs and depends on the type and stage of production of the animal to be vaccinated, cost of vaccination, expected outcome after vaccination and the type of infectious agent to be controlled [36]. Animals are vaccinated to increase productivity and profitability of livestock farmers, welfare improvement through disease prevention (similar to humans), and reduce the risk of disease transmission, particularly zoonotic diseases [36] Although veterinary vaccines have been estimated to account for less than 25 percent of the global vaccines market, their production has been increasing due to renewed demand to combat various pathogens re-emerging in the livestock industry [41]. For example, it has been noted that the pathogen MAP that causes JD can survive in water, the broader environment and through processes, including milk pasteurization, and therefore, remains present in dairy and meat products derived from infected animals, posing some human health risk [42].

Risk perception, acceptance of vaccination strategies relative to culling, and consumers’ willingness to consume meat from animals vaccinated against epidemics by stakeholders in the food system have been examined. The results show that while all stakeholders have a high preference for vaccination strategy, 60 percent agreed to consume meat from animals vaccinated against animal epidemics [43,44].

Generally, vaccines are categorized into *living* and *non-living* vaccines. The type of vaccine is a major factor that shapes its formulation and delivery. Living vaccines are divided into three main categories, including live-attenuated, inactivated (killed) and subunit vaccines. While live-attenuated vaccines comprise the weakened form of the microbe that causes the disease, which creates adaptive and long lasting immune responses [41], there is risk of possible residual virulence and reversal to pathogenic wild-type strains, with potential unintended consequences if non-target species ingest the vaccines [41].

Non-living vaccines comprising microbial antigens have been developed following advances in vaccinology and biotechnology. Although vaccines with non-living antigens are perceived to be safer relative to live or live-attenuated vaccines, they tend to be less effective and, therefore, often require the inclusion of immunological adjuvants and multiple doses to give the desired level of immunity [45]. This, therefore, translates to increased production cost and has implication for adoption by end-users. A prime example is the use of whole-cell vaccines against MAP infection. Studies (e.g., [46]) show that whole-cell vaccines only reduce symptoms and limit shedding of MAP in faeces and, therefore, are not sufficient to prevent its spread. In addition, such vaccines have been shown to cause local granulomatous lesions at the injection sites and interfere with serodiagnostic tests for MAP and bovine tuberculosis [47].

Recombinant-protein subunit vaccines with well-defined composition and produced in heterogeneous expression systems have gained more attention in recent years [41]. Subunit vaccines contain a part of the target pathogen to ensure that immune response is restricted to that component only, and produced by isolating a particular immunogenic protein from the pathogen and presenting it as an antigen on its own, which is then cloned, expressed and purified [38]. The resulting product is combined with a proper potent adjuvant to enhance immunity and used as a subunit recombinant vaccine [38].

Subunit vaccines are considered to be a safe approach due to their inability to replicate in the host [38,48]. Safety is enhanced because the vaccine contains only a part of the target pathogen, ensuring that the expected immune response is restricted to the target component alone [41]. From a compatibility perspective, an ideal subunit vaccine would allow diagnostics tests to be performed without obscuring the results.

Presently, four types of vaccines are available for the control of JD. These include live-attenuated, DNA, recombinant protein-based and subunit vaccines [49]. Studies using mice, goat and cattle models have shown advances in the development of subunit vaccines for JD and bTB. This has resulted in the identification of promising recombinant protein antigens (e.g., antigen 85 Complex A,B,C; LprG, AhpC, SodD, AhpD, MAP2698c, MAP3184, MAP3567, MAP0261c, MAP1518) that have the capacity of inducing protective immune responses and overcoming the interference in tuberculosis diagnosis tests using whole-cell based vaccines [50]. These studies highlight different techniques and vaccine candidates in developing efficacious vaccines that will enhance vaccination strategies against JD, bTB and other animal diseases. For example, [51,52] show that inclusion of Hsp70 protein in the pre-absorption stage of antibody-based assays for paratuberculosis eliminates cross-reactive antibodies and differentiates infected from vaccinated animals. In another study, the specificity of comparative tuberculin test was not interfered by subunit vaccine with candidate protein Hsp70/DDA [53,54] utilized vaccine candidate 35–kDa Membrane peptide (MMP) modified for the expression in mammalian cell to extract Cytotoxic CD8 T cell activity against *Mycobacterium avium*. Result of the study in cattle model shows prospects in developing a peptide-based vaccine that could abrogate MAP infection by removing *relA* mutant. [55] created a polyanhydride-based nanovaccine against paratuberculosis infection. Unlike the whole-cell based vaccines, the nanovaccines caused no inflammatory lesions at injection sites and induced high immune response (reduction of bacteria load) post-vaccination, an evidence of protection against JD.

For bovine tuberculosis, [56,57,58] examine the effectiveness of DNA vaccines (including DNA prime-protein boost) against tuberculosis in cattle. The results show that protective antigens—DNA encoding MPB83, HSP65 and Ag85b, respectively—showed a strong proliferative immune response, elicited protective immunity, and most importantly none of the protocols were sensitive to the intradermal tuberculin test in cattle. This suggests that DNA-based subunit vaccines can differentiate between vaccinated cattle and those infected with M. bovis.

From an efficacy point of view, the literature suggest that subunit vaccines have the potential of having a strong biological effect on the pathogens in question. However, the protection is not guaranteed to have a protracted effect. Various studies recommend the use of immuno-stimulatory compounds or adjuvants to ensure long-term protective immunity [59] Some studies suggest that booster doses may be required to enhance and extend the protective elements of the subunit vaccines to ensure there are no windows of vulnerability for livestock infection.

### 2.2. Vaccine Development

**Figure 2:**
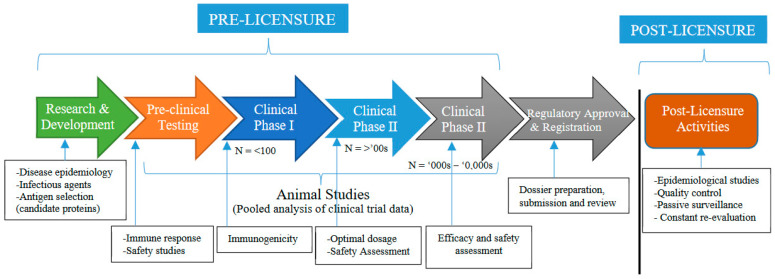
Stages in vaccine development. Source: Adapted from [61,62].

Vaccine development, whether for animals or humans, is a long costly and complex process due to increasing regulatory requirements and challenges (e.g., intellectual property) along the regulatory pathway [60]. Three major attributes expected of any vaccine by the regulatory agencies include quality, safety and efficacy. The vaccine development process involves different stages, as shown in Figure 2.

The first stage in the vaccine development process is research and development (exploratory stage). This stage involves the examination of the disease epidemiology and identification/selection of antigens (candidate proteins) that can be used to treat or prevent the disease. At the pre-clinical stage, the safety of the selected antigens is assessed in animals. The clinical development stage comprises three phases. In phase I, safety of the selected antigens is assessed with less than 100 animals. Evaluation of the immune responses takes place in phase II. Phase III involves testing the efficacy and tolerance of the vaccine on a large scale. If the tests give satisfactory results, all the data collected in the three stages are gathered and submitted to the regulatory authorities for review. Satisfactory confirmation of the safety and efficacy claims results in regulatory approval, registration (licensing), issuance of marketing authorization and product launch. Post-licensure activities include quality control, passive surveillance (monitoring the effectiveness of the vaccine) and constant re-evaluation.

## 3. Gaps in Vaccine Policies and Strategies

The slow emergence of Johne’s disease and bovine TB symptoms has contributed to the elusiveness of an effective remedy in the cattle and dairy sectors. The development of diagnostic tools, such as DIVA (Differentiating between Infected and Vaccinated Animals), that go hand-in-hand with potential vaccines could enhance vaccination control program policies. DIVA tests are used for both JD and bTB to assist farmers and testing agencies to identify infected animals and differentiate them from those that are vaccinated. The ability to develop tests that can adequately and accurately distinguish between infected and vaccinated animals is a key foundation to the safe and efficient movement of animals across borders, thus encouraging market expansion through international trade [63].

Given the perceived inefficiency of the current DIVA diagnostic tools, under European law vaccinated animals that test positive must be treated as infected animals. If an animal in a herd tests positive, the entire herd will be put under movement restrictions and tested repeatedly using both the tuberculin skin test and postmortem examinations, until the herd is free of infection [63]. The economic burden on farmers from this inability to distinguish between vaccinated and infected animals, and the duration of movement restrictions, is thus substantial.

Using a country-specific example to highlight the limitations of vaccination control programs for bTB, the disease continues to exist in the United Kingdom despite an intensive and costly control program [63]. The authors posit that although vaccination can provide some protection to cattle, it is currently illegal within the European Union (EU) due to the interaction of BCG (Bacillus Calmette-Guérin) with the action of the established tuberculin skin test used to screen for bTB. The EU has signaled that changes in any legislation would require field validation of BCG as a supplement to existing controls. This current policy vacuum will need to be filled with the creation of more effective DIVA tests that would complement current EU infection identifier tests.

**Figure 3:**
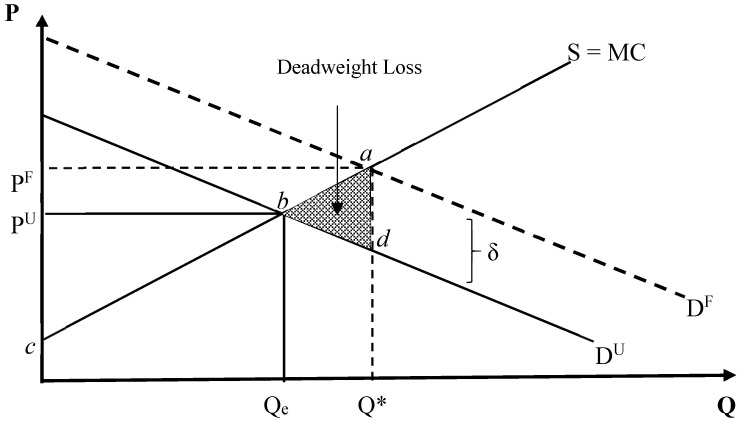
Figure showing the effect of mediocre Differentiating between Infected and Vaccinated Animals (DIVA) tests for Johne’s disease (JD) and bovine tuberculosis (bTB). The symbol “*” distinguishes the equilibrium quantity, Qe, from the new quantity, Q*.

The need for new diagnostic tests to enhance viability of vaccination against bovine tuberculosis has been suggested [63]. However, the number of false positives from these tests must be below 15 for every 10,000 cattle tested. Researchers at the University of Cambridge and the UK Animal and Plant Health Agency used mathematical modelling to show that the key factor is the specificity of the test in terms of the number of animals that are not infected and test negative, rather than the efficacy of a vaccine that will determine the feasibility of any test. Creating diagnostic tools (e.g., subunit vaccines) that are compatible with international trade and cross-border transactions should translate into increased vaccine demand and usage. Figure 3 shows a dual situation where the vaccine market is dependent on the current diagnostic tests that cannot differentiate between infected and vaccinated animals.

In Figure 3, *P* is the price of vaccines while *Q* represents the quantity of vaccines. *D^U^* is the demand curve representing the status quo of diagnostic testing with uncertainty while *D^F^* depicts the demand curve for diagnostic tests that can effectively differentiate between infected and vaccinated animals. *S* is the supply curve. Q_e_ and Q* are the equilibrium volumes at market clearing prices P^u^ and P*, representing the two different demand conditions. As a result of the current diagnostic uncertainty, the market under-provides vaccines as cross-border testing agencies cannot differentiate infected animals from those that are vaccinated. With the availability of an effective DIVA test, vaccine demand could be higher as credible mechanisms would be in place to identify infected versus vaccinated animals as showed by the demand curve, *D^F^*.

In the absence of an effective and robust diagnostic test, resources are inefficiently allocated, thereby leading to a deadweight loss (DWL) represented by area (triangle) *‘abd’*. The surplus to DIVA (subunit) vaccine producer at price *P^F^* is given by the area ‘*P^F^ac*’ while that of whole-cell based vaccines (status quo) is the area ‘*P^U^bc*’. Hence, change in producer surplus (*P^F^ac–P^U^bc*) is given by the area ‘*P^F^abP^U^*’. The difference between *D^F^* and *D^U^*, represented by *δ*, could be described as direct loss (in terms of revenue) to vaccine production companies resulting from reduced quantity (*Qe*) of vaccines demanded and sold at price (*P^U^*). This is given by (P^F^Q*–P^U^Q_e_). This also translates to reduced revenue to livestock producers who may have vaccinated their animals and enjoy the high price, *P^F^*, with low possibility of disease transmission but cannot access international markets given the unreliability of the current diagnostic methods.

### Vaccine Commercialization

Determining the best mechanism to achieve optimal market access for vaccines, given the heterogeneity and commoditization of the global vaccine market, has been identified as a major challenge in the commercialization of vaccines globally [64]. Recently, the vaccine sector has experienced a paradigm shift—moving from the traditional model primarily made-up of “pediatric vaccines” used to avert several bacterial and viral infections, to a more sophisticated pharmaceutical model that attracts premiums and is propelled by emerging new technological innovations resulting from investments in research and development [64]. BioProcess International’s description of the new vaccine development approach captures the current development of a subunit vaccine that can combat production disruptive diseases such as JD and bTB.

In practice, successful commercialization of a vaccine largely depends on the outcome of the epidemiological surveillance and/or affirmation of the vaccine as well as its value proposition [64]. A range of factors define market acceptability of a vaccine, including: *accessibility,* which depends on the available infrastructure and procurement mechanism; *availability,* or the capacity of the producer or supplier to produce in sufficient quantity that will meet perceived need by end-users; and *affordability,* which is contingent on the prescribed doses, vaccine administration and development status of the host country [64]. Other factors include compatibility with other vaccines, ease of administration—whether the vaccine requires a follow-dose—timing and whether the vaccine is a novel product.

Bioniche Life Sciences, the organization charged with the marketing and distribution of the *E. coli* vaccine Econiche, chose to enter the market through public deliver, by pushing for the government of Canada to make cattle vaccination mandatory to address the public health safety concerns posed by *E. coli*. This strategy, however, was not successful, as government intervention requires cost/benefit conditions to be justified.

Scholars (e.g., [65]) highlight new strategies currently utilized in the commercialization process to expand the product into broader markets in developing countries. Specifically, a variety of new procurement and supply strategies are being explored, one which involves an advance-purchase contract that would incentivize market purchase by donors when a new vaccine is released in the market. It is argued that guaranteed advance purchases would reduce uncertainty, overcome any hold-up or public food concerns, and increase the size of the market by accelerating a vaccine’s introduction. Furthermore, this in turn may trigger a trickle-down effect whereby the reduced risk and increased incentives could attract private investment in developing and supplying vaccines more rapidly. Such an approach could also allow the manufacturer and the purchasing entity to structure a price curve that would allow for high prices to be charged during the early donor-financing years, but guarantee lower prices once developing-country governments assume the responsibility of the contract [65].

Incentives for adoption and cost considerations are very important. Creation of new supply chains that exist separately from the existing conventional cattle value chain, particularly in the absence of diagnostic tools that can distinguish between infected and vaccinated animals, is ideal. However, in the current policy environment, any adopter of the vaccines would be faced with increased operational costs (vaccine purchase and administration) and differentiation costs (having to preserve the identity of vaccinated cattle), which are likely to be prohibitive. If vaccinated and non-vaccinated cattle are pooled together at different stages of the supply chain, the benefit of the cattle subunit vaccine would be diminished. In order to enhance the adoption of JD and bTB vaccines, savings realized from losses due to culling would need to be supplemented with premiums. This would require innovative supply chain governance structures to translate the value placed on the reduced risk from vaccinated cattle. One way these governance structures could emerge would be through branded-beef alliances.

The overall annual benefit-cost balance of a potential bTB vaccine will be determined by a number of factors: whether it will be compulsory to vaccinate all cattle, including neonates, or whether it will only be a voluntary scheme; whether the vaccination policy targets high-risk herds or only certain animals in individual herds; who pays for the vaccine and its delivery; and the duration of immunity and frequency of repeat vaccination costs [66].

Successful commercialization of JD and bTB subunit vaccines would trigger market expansion. Recently, there has been increasing shift in dietary patterns globally involving high consumption of meat, particularly in less developed countries [67]. The emergence of the middle class in BRIC (Brazil, Russia, India and China) nations is increasingly translating into an increase in the global demand for animal-based food products, thereby offering the opportunity for increased exports to these markets [68]. The development of innovative vaccines through genomics should make a significant contribution to the increasing global demand for meat products by ensuring reliable and economical livestock production and consumption.

**Figure 4:**
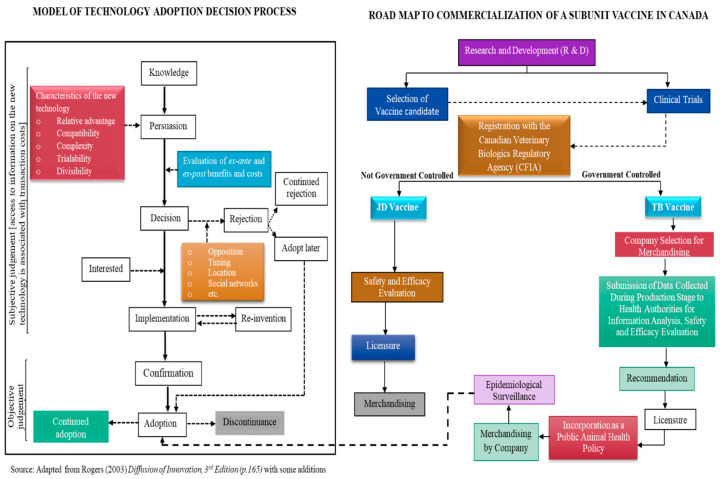
Road map to commercialization of subunit vaccines for JD and bTB (Canadian example). Source: Authors’ construction.

Subunit vaccines have different control mechanisms. Based on this, we develop different commercialization pathways for vaccines (see Figure 4) following the Canadian veterinary biologics regulatory protocol and linked this with the standard technology adoption decision process developed by Rogers (2003). (This is applicable only in Canada and may differ in other countries.)

In Canada, administration of a vaccine for JD, after production and registration (regulatory approval) is not controlled by the government. Therefore, successful commercialization would involve going through the private markets (e.g., direct sale to private companies or corporate sector). This may offer flexibility and less bureaucratic bottlenecks. One possible policy intervention would be an advance purchase contract that will guarantee purchase and reduce supply uncertainty. This would accelerate the introduction and distribution of the vaccine to different countries, thereby increasing the size of the market. This pathway could secure the incentive for private investments in vaccine production and supply by allowing for higher prices for the investor in the early years with potential lower prices in later years as argued by [65]. The downside of following this pathway for the JD vaccine is the absence of external validation of safety and quality.

In contrast, TB vaccines are currently, and for the foreseeable future, exclusively controlled by the government in Canada. Therefore, adoption will depend on whether vaccination is mandatory for all cattle or a voluntary scheme that targets only high-risk herds, who pays for the vaccine, duration of immunity, and whether there is need for booster doses

Commercialization strategies can be linked to the theory of technology adoption. End-users acceptance (adoption) and willingness to pay for subunit vaccines would be contingent on the outcome of the safety and efficacy evaluation by regulatory authorities. Recommendation by veterinarians would be enhanced if the vaccines are confirmed to be safe and efficient. Incorporating vaccines as part of animal health policy following positive outcomes of epidemiological surveillance will create incentives for continued adoption, particularly by the private sector.

## 4. Role of Livestock Farmers and Regulators in Disease Management, Policy Choice and Incentives

Given the increasing volume of cross-border trade in animal products, human and animal health risks associated with infectious diseases, and increasing clusters and concentration of livestock production, there is need for adequate livestock disease management [69]. Farmers are the primary decision makers in animal disease management. Farmers are always the front line of defense against any outbreaks, as they stand to lose the most. They always have clear incentives to prevent livestock diseases and, therefore, seek to balance marginal benefits of efforts against the marginal costs [69]. Availability of producer market incentives ought to be an integral part of livestock disease management, as absence of any incentives would reduce producers’ willingness to actively engage in prevention and/or control of animal diseases such as JD and bTB. The social costs associated with animal disease outbreak, therefore, necessitate the alignment of private market incentives with public policy goals related to animal diseases [69,70].

Farmer engagement in livestock disease management is contingent on their knowledge about the disease in question, perceived risks, alternate prevention techniques, available treatment, their costs, and market reaction, among others. Producers need to be confident in their ability to recognize clinical signs of animal diseases. This drives their propensity to monitor and respond in a timely way to emergent disease outbreaks [71].

Spillover effects (externalities), the public good nature of animal diseases with the associated ‘free-rider’ problem, information asymmetry, coordination problems, income inequality, among others, clearly justify government intervention in animal disease control [72]. Reports have shown that animal disease outbreaks in various jurisdictions resulted in substantial financial losses that put some (especially small-scale) farmers out of business. In addition, some existing disease prevention and control strategies have not been overly effective, which creates uncertainties among livestock farmers. Investment in R&D that creates new technologies that would be effective and also minimize costs of disease prevention and control could trigger a robust response to animal disease prevention and control by farmers [73].

One may argue that controlled management practices, which may be expensive at the early stages of implementation but pay off over time, would be cost-effective compared to vaccination. Some studies (e.g., [74]) examine the effects of vaccination versus management protocols in animal disease control. Although vaccines may increase costs, studies have shown that efficacious vaccines optimize disease control. Early vaccination protects animals from infection and reduces the spread of a disease after confirmation of infection or exposure, thereby minimizing the impact on productivity [75]. However, complementing good management practices with vaccination could translate to savings for both farmers and regulators in the event of a disease outbreak.

A key question is: what policy frameworks can regulators adopt to ensure that livestock farmers are best served and incentivized to participate in effective livestock disease management? A number of strategies could enhance farmers’ involvement in the management and control of livestock diseases including: creation of compensation schemes for animal diseases; and encouraging effective communication (e.g., field visits, TV commercials, radio programs) and social connectivity (e.g., agricultural shows) amongst farmers, and through influencers that serve as critical pathways or sources of information. In addition, education of farmers on animal disease risks and their potential effects beyond the farm, training for capacity building, providing incentives on early reporting of disease, inducing sufficient effort by farmers to prevent disease by shifting part of the risk to them (usually farmers have low incentives to adopt disease management strategies if the government provides everything for free and bears the whole risk; sharing the risks would encourage farmers to engage in disease prevention and control) through less-than-full compensation of losses, or differentiation of payments according to individual risk profiles potentially would create incentive for increased participation in disease management [69].

The results of some studies (e.g., [76]) showed that information used for animal disease surveillance is often provided by farmers, thereby suggesting the need for involvement of farmers (or farmer associations) in the planning and implementation of animal health policies. This will ensure that government policies and programs address farmers’ needs and create a win–win situation [76].

## 5. The Role for Public–Private Partnership (PPP)

Technological innovation is an important factor that drives sustained growth in productivity across sectors, and its success is often contingent on the extent of collaboration between different actors in the innovation space [77]. Developing modern research and disseminating research output in a global environment requires effective collaboration between the public and private sectors, organization of co-operations at different levels, coordinating national and international policies, promoting networking between teams and increasing the mobility of individuals and ideas [78].

PPPs involve inter-sectoral collaborations in which two or more parties work together and share responsibilities, resources, risks, benefits and accountability under a contractual agreement [79]. For a successful PPP, as noted by [80], there must be a common interest between the public and private sector parties, positive benefit–cost ratios to PPP and potential for synergy.

In principle and reality, the public sector is responsible for providing funding for activities related to the prevention and control of endemic animal diseases, perhaps because of the magnitude of social costs associated with disease outbreaks. However, in some cases, the public sector has limited capability to provide the needed institutions infrastructure, logistics and monitoring to actualize this objective. Where the public sector is solely responsible, such efforts, sometimes, may not get to small-scale farmers in remote areas. Hence, relying on the public sector alone may result in suboptimal disease prevention and control. This, therefore, necessitates alliances between the public sector (with the mandate of providing public goods) and the private organizations with technical expertise and needed resources to achieve optimal disease control and food security. Such combined intervention to reach a common goal has been recommended to be part of any nation’s comprehensive development framework [81], and an integral part of innovative framework and policies that will deliver efficient animal health services.

Industry-led technological development, in most cases, requires huge financial investments that can best be funded through public–private partnerships. For example, in the livestock industry, public–private partnerships could facilitate the development of new and effective tools such as vaccines and diagnostic tests for the control of animal diseases. Assisting the private sector in overcoming investment barriers could spur the development of industrial processes, products and services that otherwise would not emerge spontaneously [77]. Effective animal health policy and development and use of new vaccines may perhaps best be achieved through a coherent regulatory framework that identifies the gaps in an existing animal disease control mechanism, diagnosis or treatment, and prioritizes animal-related threats [78]. PPPs could help with that process.

In the case of bTB and JD, the PPP model could improve disease surveillance (early detection), prompt response (treatment), coordination and effective implementation of control programs and livestock farmers’ convenience. Public sector collaboration with private animal health practitioners whose operating units are close to farmers’ operations will enhance early detection and notification of bTB and JD outbreaks. Farmers with infected herds will have easy access to diagnosis and treatment centers. This will reduce disease transmission. As suggested by [82], the private sector could also manage compensation programs in the event of disease outbreak. This would incentivize farmers to engage in better management and disease control practices.

In some countries, especially low-income countries, a majority of people prefer going to private clinics either for themselves or their animals because of easy access, convenience and quality of treatment despite the costs, resulting in favorable competition with the public sector. Results of studies (e.g., [83]) show that a significant proportion of tuberculosis cases in high prevalence countries, including Uganda, India, Kenya, Pakistan, among others, were detected and treated by private practitioners.

Furthermore, the PPP model may be necessary to get adequate support for extension services for bTB and JD control. Distribution of vaccines and actual vaccinations for cattle could be collaboratively done either by the public and private partners. While the public sector takes responsibility of disease surveillance and information systems, quality control of vaccines, laboratory/diagnostic facilities and monitoring of animal movements, the private sector could administer the vaccines in coordination with public sector agencies. The two sectors could jointly carry out vigilance activities and promotion of animal health education among farmers. There is one model of this operating in Brazil for the control of Foot and Mouth Disease (FMD) in cattle [84]. In addition, disease-free status for bTB was achieved in Australia between 1989 and 1997 as a result of robust collaboration between the government and industry, as they co-funded eradication programs [85].

However, productive and innovative PPPs in animal disease prevention and control require transparency in the regulatory framework and stable and/or conducive public policy environments. Sustainable PPPs are most often found where there are relevant regulatory frameworks, policies and engagement platforms that offer win (society)–win (public)–win (private) [86].

## 6. Implications for Food Security

Food security encompasses four dimensions, including physical availability of food, economic access, utilization and stability over time [87]. Food security exists when “all people, at all times have physical, social and economic access to sufficient, safe and nutritious food that meets their dietary needs and food preferences for an active and healthy life” [88].

Livestock production is an important aspect of food production and plays a crucial role in food security. About one-third of global crop area is used to produce feed for livestock and about 75 percent of global agricultural land area is used for livestock grazing [89] Animal products make up about one-third of global nutrition. To achieve food security, sustainability of the supply of food products from livestock is essential; negative environmental impacts on livestock and crops must be adequately minimized. The effect of an animal disease on food security largely depends on: the type of disease, rate of spread, duration and severity of infection [90] the category (species) of animals it affects, whether the disease is endemic; and the impact on the affected animal.

Animal diseases, such as bTB and JD, impact negatively on animal health, welfare, productivity, and reduce potential for livestock farming intensification. While these two diseases often result in revenue losses by reducing meat quantity and quality through disposal of affected parts or the entire animal carcass deemed unfit for consumption, JD reduces milk production in ruminants as well as fertility in animals. bTB and JD infection could result in predisposition to other animal diseases. MAP infection has also been associated with reduced immune competences of infected animals, thereby exposing the animals to other diseases [21].

JD and bTB outbreaks affect food security as they often result in massive culling, premature slaughter, disruptions of food supply chains, and interruptions in cross border trade. bTB outbreaks also pose significant health risk to human populations. International trade contributes to improved food and nutrition security as it helps in balancing supply and demand. Disruptions can lead to shortages and gluts that amplified food insecurity or disrupt efficient production. From the perspective of importers, reduced barriers on live animals and livestock products’ trade for countries that are bTB and JD free will afford the consumers the opportunity of enjoying variety of animal products at affordable prices for a balanced diet. This has been shown to reduce diseases and mortality as well as improve learning ability [91].

One way of achieving sustainable livestock production and food security is through technological interventions such as vaccines that improve animal health, productivity and profitability while reducing market risks. Bringing safe, efficient and effective vaccines for bTB and JD into the system required widespread action, involving actors from the public and private sector and new partnerships.

## References

[1] Steinfeld H., Gerber P., Wassenaar T., Castel V., Rosales M., de Haan C. (2006). Livestock’s Long Shadow: Environmental Issues and Options.

[2] Popp A., Lotze-Campen H., Bodirsky B. (2010). Food consumption, diet shifts and associated non-CO_2_ greenhouse gases from agricultural production. Glob. Environ. Chang..

[3] Pokam B.D., Guemdjom P.W., Yeboah-Manu D., Weledji E.P., Enoh J.E., Tebid P.G., Asuquo A.E. (2019). Challenges of Bovine Tuberculosis Control and Genetic Distribution in Africa. Biomed. Biotechnol. Res. J..

[4] Bicknell K.B., Wilen J.E., Howitt R.E. (1999). Public policy and private incentives for livestock disease control. Aust. J. Agran. Resour. Econ..

[5] Ochieng B., Hobbs J.E. (2016). Incentives for cattle producers to adopt an E. coli vaccine: An application of best-worst scaling. Food Policy.

[6] University of British Columbia UBC Microbiologists to Use ‘Reverse Vaccinology’ to Combat Johne’s Disease, Bovine Tuberculosis. 24 November 2015. https://science.ubc.ca/news/ubc-microbiologists-use-%E2%80%98reverse-vaccinology%E2%80%99-combat-johnes-disease-bovine-tb.

[7] Ott S.L., Wells S.J., Wagner B.A. (1999). Herd-level economic losses associated with Johne’s disease on US dairy operations. Prev. Vet. Med..

[8] Garry F., Wells S., Ott S., Hansen D. (1999). Info Sheet: APHIS Veterinary Services: Who can Afford a $200 Loss Per Cow? Or Johne’s Disease—What do I Need to Know?. https://www.aphis.usda.gov/animal_health/nahms/dairy/downloads/dairy96/Dairy96_is_Johnes_1.pdf.

[9] Chiodini R.J., Van Kruiningen H.J., Merkal R.S. (1984). Ruminant paratuberculosis (johne’s disease): The current status and future prospects. Cornell Vet..

[10] Tiwari A., VanLeeuwen J.A., McKenna S.L., Keefe G.P., Barkema H.W. (2006). Johne’s disease in Canada Part I: Clinical symptoms, pathophysiology, diagnosis, and prevalence in dairy herds. Can. Vet. J..

[11] Arnoldi J.M., Hurley S. Johne’s disease in Wisconsin cattle—A survey of cullcows. Proceedings of the First International Colloquium of Research in Paratuberculosis.

[12] Whitlock R.H., Hutchinson L.T., Merkal R.S. (1985). Prevalence and economic consideration of johne’s disease in the northeastern U.S. Proc. USA Anim. Health Assoc..

[13] Gill I.J., Milner A., Wood P. (1989). The economic impact of johne’s disease in cattle in Australia. Johne’s Disease.

[14] Wilson D.J., Rossiter C., Han H.R., Sears P.M. (1993). Association of mycobacterium paratuberculosis infection with reduced mastitis, but with decreased milk production and increased cull rate in clinically normal dairy cows. Am. J. Vet. Res..

[15] Merkal R.S., Larsen A.B., Booth G.D. (1975). Analysis of the effect of inapparent bovine paratuberculosis. Am. J. Vet. Res..

[16] Raizman E.A., Fetrow J., Wells S.J., Godden S.M., Oakes M.J., Vazquez G. (2007). The association between *mycobacterium avium* ssp. *Paratuberculosis* fecal shedding or clinical johne’s disease and lactation performance on two Minnesota, USA dairy farms. Prev. Vet. Med..

[17] Palmer M.V. (2013). Mycobacterium bovis: Characteristics of Wildlife Reservoir Hosts. Transbound. Emerg. Dis..

[18] Fitzgerald S.D., Kaneene J.B. (2013). Wildlife Reservoirs of Bovine Tuberculosis Worldwide: Hosts, Pathology, Surveillance, and Control. Vet. Pathol..

[19] Palmer M.V., Wiardra J., Kanipe C., Thacker T.C. (2019). Early Pulmonary Lesions in Cattle Infected via Aerosolized Mycobacterium bovis. Vet. Pathol..

[20] Canadian Food Inspection Agency (2017). Bovine Tuberculosis. http://www.inspection.gc.ca/animals/terrestrialanimals/diseases/reportable/tuberculosis/eng/1330205978967/1330206128556.

[21] Bergen R. (2011). Keeping up with Johne’s. *Canadian Cattlemen’s Association (CCA) Magazin*. https://www.canadiancattlemen.ca/2011/01/24/keeping-up-with-johnes/.

[22] Matthews L., Reeve R., Gally D.L., Low J.C., Woolhouse M.E.J., McAteer S.P., Locking M.E., Chase-Topping M.E., Haydon D.T., Allison L.J. (2013). Predicting the public health benefit of vaccinating cattle against *Escherichia coli* O157. Proc. Natl. Acad. Sci. USA.

[23] United States Department of Agriculture Animal and Plant Inspection Service (2014). Questions and Answers: Bovine Tuberculosis. https://www.aphis.usda.gov/publications/animal_health/content/printable_version/faq_bovine_tb_.pdf.

[24] Scot P. (2009). Johne’s Disease Paratuberculosis. http://www.nadis.org.uk/disease-a-z/cattle/johnes-disease-paratuberculosis/.

[25] Spickler A.R. (2019). Zoonotic Tuberculosis. http://www.cfsph.iastate.edu/Factsheets/pdfs/bovine_tuberculosis.pdf.

[26] Medeiros L.S., Marrasi C.D., Figueiredo E.E.S., Leite J., Ferreira A.M.R., Lilenbaum W. (2012). Assessing the histopathology to depict the different stages of bovine tuberculosis infection in a naturally infected herd. Pesquisa Veterinária Brasileira.

[27] Gormley E., Corner L.A., Costello E., Rodriguez-Campos S. (2014). Bacteriological diagnosis and molecular strain typing of *Mycobacterium bovis* and *Mycobacterium caprae*. Res. Vet. Sci..

[28] Monaghan M.L., Doherty M.L., Collins J.D., Kazda J.F., Quinn P.J. (1994). The tuberculin test. Vet. Microbiol..

[29] Wood P.R., Rothel J.S. (1994). In vitro immunodiagnostic assays for bovine tuberculosis. Vet. Microbiol..

[30] Varello K., Pezzolato M., Mascarino D., Ingravalle F., Caramelli M., Bozzetta E. (2008). Comparison of histologic techniques for the diagnosis of bovine tuberculosis in the framework of eradication programs. J. Vet. Diagn. Investig..

[31] National Research Council (2003). Diagnosis and Control of Johne’s Disease. Committee on Diagnosis and Control of Johne’s Disease. http://www.nap.edu/catalog/10625.html.

[32] Hendrick S., Douma D. (2006). Literature Review of Johne’s Disease in Beef Cattle. Western College of Veterinary Medicine, University of Saskatchewan. http://www.beefresearch.ca/files/pdf/johnes-disease-lit-review-hendrick.pdf.

[33] Sockett D.C., Carr D.J., Collins M.T. (1992). Evaluation of conventional and radiometric fecal culture and a commercial DNA probe for diagnosis of mycobacterium paratuberculosis infections in cattle. Can. J. Vet. Res..

[34] Whitlock R.H., Buergelt C. (1996). Preclinical and clinical manifestations of paratuberculosis (including pathology). Vet. Clin. N. Am. Food Anim. Pract..

[35] Bourne D.C. (1997). Disease and Mortality in Bennett’s Wallabis (*Macropus rufogriseus rufoseus*) at Whipsnade Wild Animal Park, with Special Reference to Toxoplasmosis. Ph.D. Thesis.

[36] Meeusen E.N., Walker J., Peters A., Pastoret P.P., Jungersen G. (2007). Current status of veterinary vaccines. Clin. Microbiol. Rev..

[37] Shryock T.R. (2004). The Future of anti-infective products in animal health. Nat. Rev. Microbiol..

[38] Lee N.-H., Lee J.-A., Park S.-Y., Song C.-S., Choi I.-S., Lee J.-B. (2012). A review of vaccine development and research for industry animals in Korea. Clin. Exp. Vaccine Res..

[39] Scheerlinck J.P., Greenwood D.L. (2006). Particulate delivery systems for animal vaccines. Methods.

[40] Sharma S., Hinds L.A. (2012). Formulation and delivery of vaccines: Ongoing challenges for animal management. J. Pharm. Bioallied Sci..

[41] Wang M., Jiang S., Wang Y. (2016). Recent advances in the production of recombinant subunit vaccines in pichia pastoris. Bioengineered.

[42] Hermon-Taylor J. (2009). Mycobacterium avium subspecies paratuberculosis, crohn’s disease and the doomsday scenario. Gut Pathog..

[43] Zingg A., Siegrist M. (2011). Lay people’s and experts’ risk perception and acceptance of vaccination and culling strategies to fight animal epidemics. J. Risk Percept..

[44] Zingg A., Siegrist M. (2012). People’s willingness to eat meat from animals vaccinated against epidemics. Food Policy.

[45] Roth J.A., Henderson L.M. (2001). New technology for improved vaccine safety and efficacy. Vet. Clin. N. Am. Food Anim. Pract..

[46] Rosseels V., Huygen K. (2014). Vaccination against paratuberculosis. Exp. Rev. Vaccines.

[47] Nedrow A.J., Gavalchin J., Smith M.C., Stehman S.M., Maul J.K., McDonough S.P., Thonney M.L. (2007). Antibody and skin-test responses of sheep vaccinated against johne’s disease. Vet. Immunol. Immunopathol..

[48] Redding L., Weiner D.B. (2009). DNA vaccines in veterinary use. Exp. Rev. Vaccines.

[49] Shanmugasundaram K., Bhupendra N.T. (2018). Johne’s Disease Vaccines Past, Present and Future. Adv. Biotechnol. Microbiol..

[50] Park H.T., Yoo H.S. (2016). Development of Vaccines for *Mycobacterium avium* Subsp. Paratuberculosis Infection. Clin. Exp. Vaccines Res..

[51] Santema W., Hensen S., Rutten V., Koets A. (2009). Heat Shock Protein 70 Subunit Vaccination against Bovine Paratuberculosis does not Interfere with Current Immunodiagnostic Assays for Bovine Tuberculosis. Vaccine.

[52] Santema W., Overdijk M., Barends J., Krijgsveld J., Rutten V., Koets A. (2009). Searching for Proteins of Mycobacterium avium subspecies Paratuberculosis with Diagnostic Potential by Comparative Qualitative Proteomic Analysis of Mycobacterial Tuberculins. Vet. Microbiol..

[53] Santema W., Rutten V., Koets A. (2011). Bovine Paratuberculosis: Recent Advances in Vaccine Development. Vet. Q..

[54] Franceschi V., Mahmoud A.H., Abdellrazeq G., Tebaldi G., Macchi F., Russo L., Fry L.M., Elnaggar M., Bannantine J., Park K.T. (2019). Capacity to Elicit Cytotoxic CD8 T Cell Activity Against Mycobacterium avium subsp. paratuberculosis is Retained in a Vaccine Candidate 35 kDa Peptide Modified for Expression in Mammalian Cells. Front. Immunol..

[55] Thukral A., Ross K., Hansen C., Phanse Y., Narasimhan B., Steinberg H., Talaat A.M. (2020). A Single Dose Polyanhydride-Based Nanovaccine against Paratuberculosis Infection. NPJ Vaccines.

[56] Chambers M.A., Vordermeier H., Whelan A., Commander N., Tascon R., Lowrie D., Hewinson R.G. (2000). Vaccination of Mice and Cattle with Plasmid DNA Encoding the *Mycobacterium bovis* Antigen MPB83. Clin. Infect. Dis..

[57] Vordermeier H.M., Lowrie D.B., Hewinson R.G. (2003). Improved Immunity of DNA Vaccination with Mycobacterial HSP65 against Bovine Tuberculosis by Protein Boosting. Vet. Microbiol..

[58] Teixeira F.M., Teixeira H.C., Ferreira A.P., Rodrigues M.F., Azevedo V., Macedo G.C., Oliveira S.C. (2006). DNA Vaccine Using, *M. bovis* Ag85B Antigen Induces Partial Protection against Experimental Infection in BALB/c mice. Clin. Vaccine Immunol..

[59] Moyle P.M., Toth I. (2013). Modern subunit vaccines: Development, components, and research opportunities. ChemMedChem.

[60] Stevens H., Debackere K., Goldman M., Mahoney R.T., Stevens P., Huys I. (2017). Vaccines: Accelerating Innovation and Access. Global Challenges Report, World Intellectual Property Organization (WIPO). https://www.wipo.int/publications/en/details.jsp?id=4224.

[61] Di Pasquale A., Preiss S., Da Silva F.T., Garcon N. (2015). Vaccine adjuvants: From 1920 to 2015 and beyond. Vaccines.

[62] British Columbia Centre for Disease Control (2009). Vaccine Development and Licensing. http://www.bccdc.ca/search?k=stages%20in%20vaccine%20development.

[63] Conlan A.J.K., Pollock E.B., McKinley T.J., Mitchell A.P., Jones G.J., Vordermeier M., Wood J.L.N. (2015). Potential benefits of cattle vaccination as a supplementary control for bovine tuberculosis. PLoS Comp. Biol..

[64] BioProcess International (2008). Global Vaccine Commercialization. BPI’s State of the Industry: Perspectives from Inside the Bio-Manufacturing Industry. http://www.bioprocessintl.com/manufacturing/monoclonal-antibodies/global-vaccine-commercialization-183986/.

[65] Batson A. (2005). The problems and promise of vaccine markets in developing countries. Health Aff..

[66] House of Commons Environment, Food and Rural Affairs Committee (2013). Vaccination against Bovine TB.

[67] World Health Organization Global and Regional Food Consumption Patterns and Trends: Availability and Changes in Consumption of Animal Products. https://www.who.int/nutrition/topics/3_foodconsumption/en/index4.html.

[68] Ludu J.S., Plastow G.S. (2013). Livestock and the promise of genomics. Genome.

[69] Organisation for Economic Co-Operation and Development (2017). Producer Incentives in Livestock Disease Management.

[70] Sumner D.A., Bervejillo J.E., Jarvis L.S. (2005). Public policy, invasive species and animal disease management. Int. Food Agribus. Manag. Rev..

[71] Wright B., Jorgensen B., Smith L. (2016). Development of Behaviour Change Strategies for Animal Disease Surveillance and Reporting. Behaviour Works Australia. http://www.ava.com.au/sites/default/files/AVA_website/pdf/BWA-Final-Report-Animal-Disease-Surveillance-September-2016_0.pdf.

[72] Ramsay G., Philip P., Riethmuller P. (1999). The economic implications of animal diseases and disease control at the national level. In the economics of animal disease control, coordinated by B.D. Perry. Off. Int. Des. Epizoot. Sci. Tech. Rev..

[73] Wolf R., Clement F., Barkema H.W., Orsel K. (2014). Economic evaluation of participation in a voluntary Johne’s disease preven-tion and control program from a farmer’s perspective—The Al-berta Johne’s Disease Initiative. J. Dairy Sci..

[74] Coloner M.A., Margalida A., Fraile L. (2020). Vaccination is a Suitable Tool in the Control of Aujeszky’s Disease Outbreaks in Pig Using a Population Dynamics P System Model. Animals.

[75] Andre F.E., Booy R., Block H.L., Clemens J., Datta S.K., John T.J., Lee B.W., Lolekha S., Peltola H., Ruff T.A. (2008). Vaccination Greatly Reduces Disease, Disability, Death and Inequality Worldwide. Bull. World Health Organ..

[76] Grace D., Jost C., Macgregor-Skinner G., Mariner J.C. Participation of small farmers in animal health programmes. Proceedings of the Conf. OIE.

[77] Shivakumar S. (2002). Innovation and the Role of Public-Private Partnerships in the Knowledge-Based Economy.

[78] Organisation for Economic Co-Operation and Development (2004). Public-Private Partnerships for Research and Innovation: An Evaluation of the Dutch Experience.

[79] (2017). World Bank PPP Reference Guide.

[80] Hartwich F., Gonzalez C., Vieira L.F. (2005). Public–Private Partnerships for Innovation-Led Growth in Agrichains: A Useful Tool for Development in Latin America? ISNAR Discussion Paper 1.

[81] Nishtar S. (2004). Public-Private ‘Partnerships’ in Health—A Global Call for Action. Health Res. Policy Syst..

[82] Delgado C., Narrod C., Tiongco M., Barros G., Catelo M., Costales A., Mehta R., Naranong V., Poapongsakorn N. (2008). Determinants and Implications of the Growing Scale of Livestock Farms in Four Fast-Growing Developing Countries.

[83] Uplekar M., Raviglione M., Pathnia V. (2001). Private practitioners and public health: Weak links in tuberculosis control. Lancet.

[84] Dubois R., Moura J.A. (2004). La lutte contre la fièvre aphteuse au Brésil: La participation du secteur privé. Rev. Sci. Tech. Off. Int. Epizoot..

[85] Black P.F. (2012). Good governance of animal health system and public-private partnerships: An Australian case study. Rev. Sci. Off. Int. Epizoot..

[86] Thevasagayam S. Public-private partnerships expectations of private sector partners for international animal health and livestock sector development programmes. Proceedings of the 85th General Session of OIE.

[87] Food and Agriculture Organization (2008). An Introduction to Basic Concepts of Food Security. www.gao.org/3/al936e/al936e00.pdf.

[88] (2012). Global Strategic Framework for Food Security and Nutrition. First Version. http://www.fao.org/docrep/meeting/026/ME498E.pdf.

[89] Kastner T., Rivas M.J.I., Koch W., Nonhebel S. (2012). Global changes in diets and the consequences for land requirements for food. Proc. Natl. Acad. Sci. USA.

[90] Fitzpatrick J.L. (2013). Global food security: The impact of veterinary parasites and parasitologists. Vet. Parasitol..

[91] Whaley S., Sigman M., Bwibo N., Guthrie D., Weiss R.E.S., Murphy S., Alber S. (2002). The Impact of Dietary Intervention on the Cognitive Development of Kenyan Schoolchildren.

